# Ruptured Cerebral Arteriovenous Malformation Initially Managed As Presumed Eclampsia: A Diagnostic Challenge Following Initial Management in a Resource-Limited Setting

**DOI:** 10.7759/cureus.113452

**Published:** 2026-07-27

**Authors:** Mirza Muflihul Haque, Debashish Banik, Mahbub Nisat Ibtasam, Shihan Mahmud R Huq, Md. Shafiqul Islam, Shahzadi Sayeeda Tun Nessa

**Affiliations:** 1 Critical Care Medicine, Square Hospital Ltd., Dhaka, BGD; 2 Critical Care Medicine, Hi-Care General and Specialized Hospital, Dhaka, BGD; 3 Neurology, Shaheed Suhrawardy Medical College and Hospital, Dhaka, BGD; 4 Medicine, Square Hospital Ltd., Dhaka, BGD; 5 Neurosurgery, Dhaka Medical College and Hospital, Dhaka, BGD; 6 Internal Medicine and Critical Care, Hi-Care General and Specialized Hospital, Dhaka, BGD

**Keywords:** avm rupture, digital subtraction angiography (dsa), life-threatening intracranial hematoma, new-onset seizure, pre-eclampsia

## Abstract

Seizures occurring during late pregnancy frequently raise immediate concern for eclampsia. However, persistent neurological deterioration despite definitive obstetric management should prompt consideration of alternative intracranial pathology. Cerebral arteriovenous malformations (AVMs) are uncommon vascular lesions that may rarely present during pregnancy with intracerebral hemorrhage and seizures.

We report the case of a 31-year-old gravida 3 para 2 woman at 36 weeks of gestation with a history of two previous uncomplicated lower-segment cesarean sections who presented with generalized tonic-clonic convulsions followed by depressed consciousness (Glasgow Coma Scale score E2V1M2) and a blood pressure of 160/90 mmHg. Presumed eclampsia was diagnosed, and emergency lower-segment cesarean section was performed. Despite delivery, seizures persisted with poor neurological recovery. Computed tomography of the brain demonstrated a large left intracerebral hemorrhage with significant mass effect and midline shift, necessitating emergency decompressive craniectomy and hematoma evacuation. As neurological improvement remained unsatisfactory, further vascular evaluation was undertaken. Computed tomography angiography identified a cerebral AVM supplied predominantly by branches of the left middle cerebral artery, with venous drainage through the superior anastomotic vein (vein of Trolard) into the superior sagittal sinus. Digital subtraction angiography confirmed the diagnosis, and endovascular embolization achieved approximately 85% reduction of the AVM nidus. The patient remained seizure-free, demonstrated progressive neurological recovery, and was discharged with a Glasgow Coma Scale score of 15. Follow-up showed independent ambulation, improvement in motor function, and no recurrent neurological events.

This case highlights the diagnostic challenges encountered in resource-limited settings, where eclampsia is frequently the initial working diagnosis for seizures during late pregnancy. Failure of neurological recovery in a young adult after appropriate obstetric management should prompt urgent neuroimaging and consideration of alternative intracranial pathology. Definitive diagnosis followed by multidisciplinary management resulted in progressive neurological improvement.

## Introduction

Eclampsia is one of the leading causes of maternal morbidity and mortality worldwide. It typically presents with new-onset generalized tonic-clonic seizures during pregnancy or the postpartum period in women with hypertensive disorders of pregnancy [1]. Although delivery is considered the definitive treatment, persistent seizures or delayed neurological recovery after delivery should prompt clinicians to consider diagnoses other than eclampsia.

Several neurological conditions can present in a similar manner, including posterior reversible encephalopathy syndrome, cerebral venous sinus thrombosis, ischemic stroke, intracranial hemorrhage, aneurysmal rupture, and other structural cerebrovascular disorders [2]. Failure to recognize these conditions may delay appropriate diagnosis and treatment, potentially leading to poor neurological outcomes.

Cerebral arteriovenous malformations (AVMs) are congenital vascular abnormalities in which arteries communicate directly with veins without an intervening capillary bed [3,4]. Although many AVMs remain asymptomatic, rupture may present with intracerebral hemorrhage, seizures, or focal neurological deficits. Whether pregnancy itself increases the risk of AVM rupture remains controversial, but the physiological and hemodynamic changes of pregnancy have been proposed as possible contributing factors [4].

We report a case of a ruptured cerebral AVM that initially presented as presumed eclampsia. Persistent neurological deterioration despite emergency cesarean delivery and life-saving decompressive craniectomy prompted further vascular evaluation, ultimately leading to the diagnosis of the underlying AVM.

## Case presentation

A 31-year-old gravida 3 para 2 woman at 36 weeks of gestation with a history of two previous uncomplicated lower-segment cesarean sections, the most recent three years earlier, presented to an outside hospital with sudden-onset generalized tonic-clonic convulsions followed by depressed consciousness. The current pregnancy had been uneventful until presentation. On arrival, her blood pressure was 160/90 mmHg and neurological examination revealed a Glasgow Coma Scale score of E2V1M2.

Given the advanced gestational age, generalized seizures, elevated blood pressure (160/90 mmHg), and depressed consciousness, the patient was managed as presumed eclampsia. A 4 g intravenous loading dose together with 10 g intramuscular magnesium sulfate (5 g in each buttock) was administered, followed by 5 g intramuscularly every four hours according to the Pritchard regimen. An emergency lower-segment cesarean section was performed because delivery is the definitive treatment for presumed eclampsia. A healthy live infant was delivered without immediate neonatal complications. Despite delivery, persistent convulsions and severely depressed consciousness continued. Because of persistent seizures and a Glasgow Coma Scale score of E2V1M2, the patient was endotracheally intubated and mechanically ventilated.

Initial laboratory investigations (Table 1) did not reveal a metabolic abnormality sufficient to explain the neurological presentation.

**Table 1:** Initial laboratory investigations ALT: alanine aminotransferase; aPTT: activated partial thromboplastin time; K: potassium; Na: sodium; PT/INR: prothrombin time/international normalized ratio; Trop-I: cardiac troponin I; WBC: white blood cell count

Test parameters	During first admission	Normal range
WBC	9,700	4000-10,000 cells/cmm
Hemoglobin	11.4 g/dL	11.5-15.5 g/dL
Platelet	350	150-450/cmm
ALT	16	< 50 U/L
Creatinine	0.85	0.60-1.40 mg/dL
Na	136	135-145 mmol/L
K	4.4	3.5-5 mmol/L
Serum Albumin	2.92	3.40-5.40 g/dL
D-Dimer	1.5	0.50 µg/mL
PT/INR	14/1.18	10.7-13.6 seconds/0.8-1.2
aPTT	36	28-36 seconds
Trop-I	0.012	<0.034

**Figure 1:**
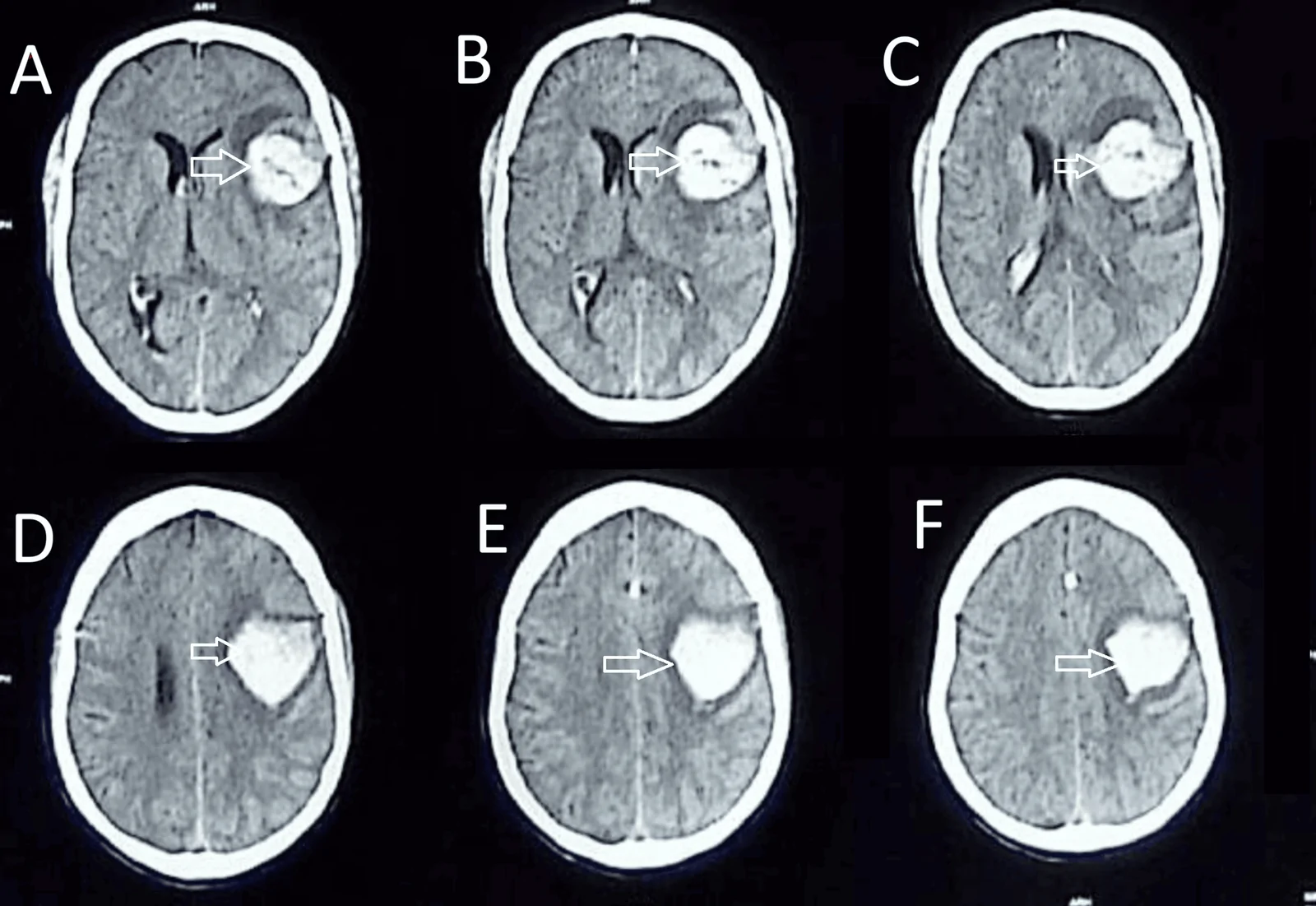
Initial non-contrast CT brain Sequential axial images (A-F) demonstrate a large left fronto-temporo-parietal lobar hemorrhage (white arrows) with surrounding edema.

Urgent neuroimaging was performed. Non-contrast computed tomography of the brain demonstrated a large left-sided fronto-temporo-parietal lobar hemorrhage with significant mass effect and midline shift (Figure 1).

Due to the life-threatening nature of the hemorrhage, the patient underwent emergency decompressive craniectomy with hematoma evacuation at the referring hospital. The procedure was performed based on the non-contrast CT findings as computed tomography angiography (CTA) was not available at that facility. No underlying vascular malformation was suspected during the initial surgery. The bone flap was not replaced, and cranioplasty was planned following neurological recovery.

**Figure 2:**
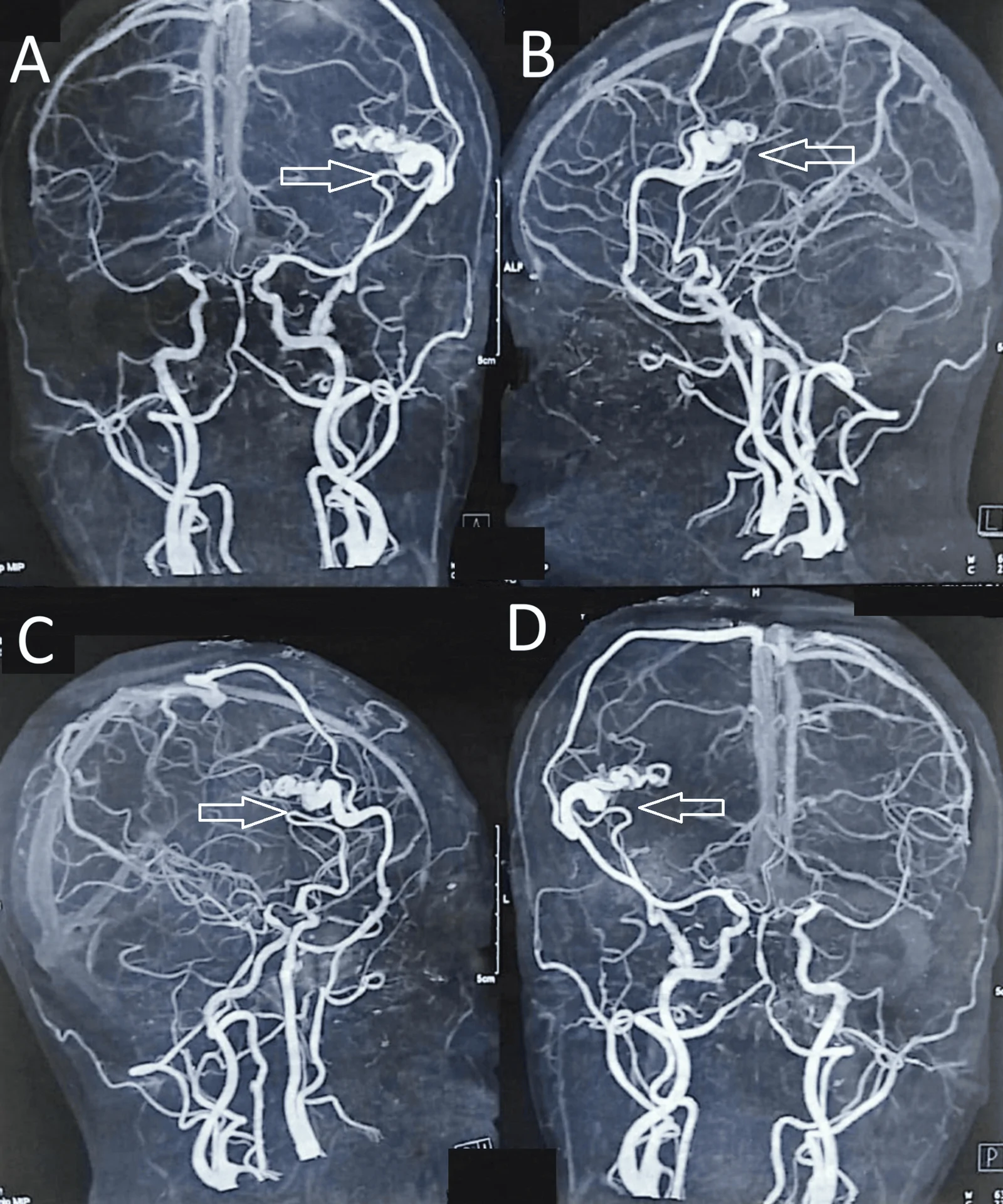
Computed tomography angiography demonstrating the cerebral AVM (A) Coronal view. (B) Sagittal view. (C) Oblique view. (D) Coronal venous reconstruction. White arrows indicate the AVM nidus. AVM, arteriovenous malformation

Although surgical decompression relieved the mass effect, neurological recovery remained limited. Mechanical ventilation was continued for five days. Following extubation, the patient regained consciousness and was able to communicate despite expressive aphasia. However, persistent expressive aphasia, right-sided weakness, and recurrent seizures, together with her young age, absence of known cerebrovascular risk factors, and the presence of a large lobar intracerebral hemorrhage, raised suspicion of an underlying structural cerebrovascular lesion. She was discharged from the referring hospital on oral levetiracetam for seizure prophylaxis and referred to a higher tertiary center for CTA. CTA revealed a cerebral AVM supplied predominantly by branches of the left middle cerebral artery, with venous drainage through the superior anastomotic vein (vein of Trolard) into the superior sagittal sinus (Figure 2).

Following the CTA findings at the second hospital, the patient was transferred to our specialized neuroscience center for definitive neurovascular management. The initial treating hospital had facilities for emergency obstetric care, non-contrast CT imaging, and hematoma evacuation but lacked access to CTA, digital subtraction angiography (DSA), and endovascular neurointerventional services. Because these specialized neurovascular services are available only at a limited number of tertiary referral centers in Bangladesh, referral to our center was required for definitive vascular evaluation and endovascular treatment.

**Figure 3:**
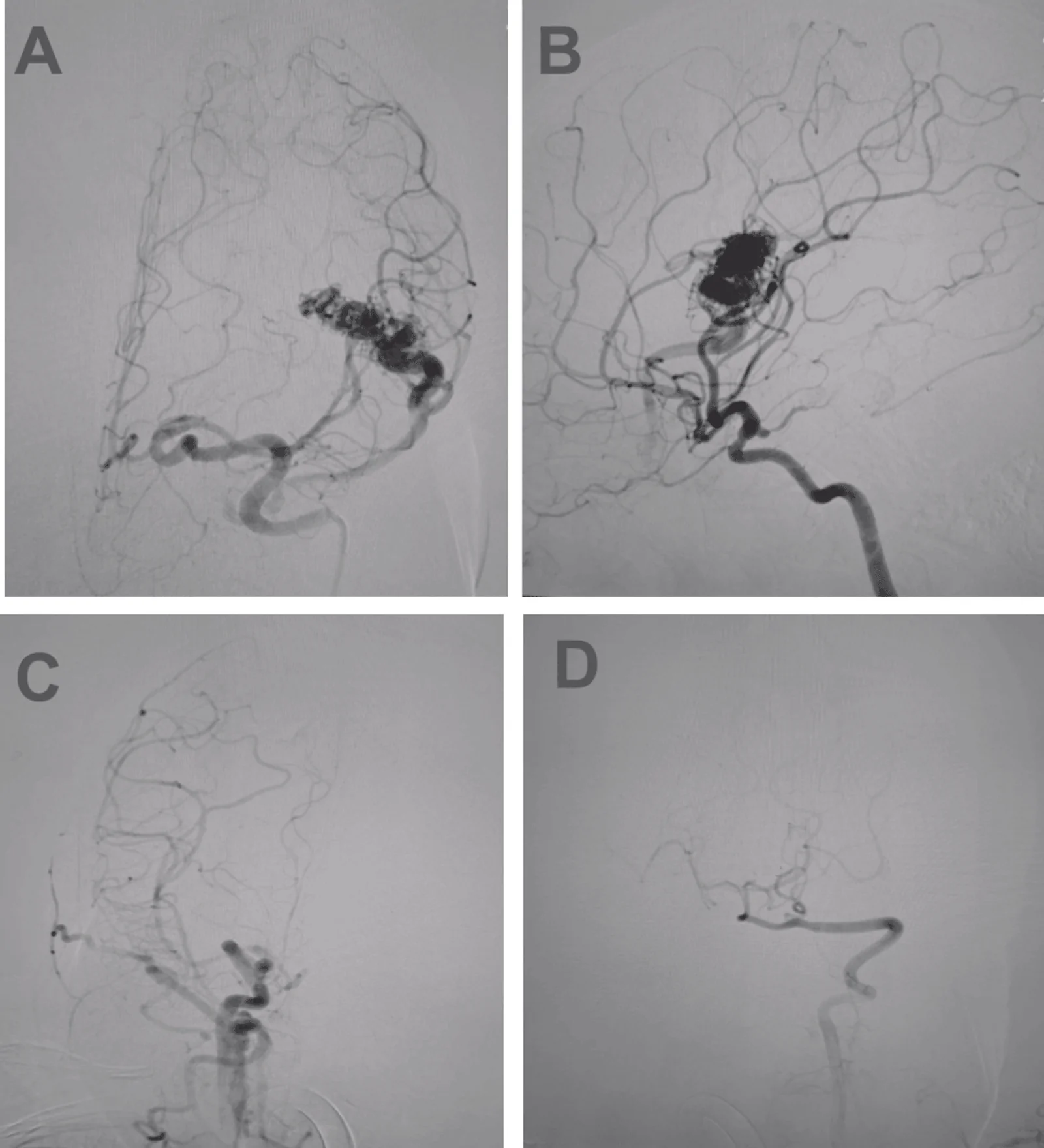
Digital subtraction angiography before and after endovascular embolization of the cerebral arteriovenous malformation (A) Pre-embolization anteroposterior projection demonstrating a left parietal AVM supplied predominantly by the superior division of the left middle cerebral artery. (B) Pre-embolization lateral projection demonstrating the AVM nidus with early arteriovenous shunting. (C) Post-embolization anteroposterior projection demonstrating marked reduction in AVM nidus opacification following Lava 18 embolization. (D) Post-embolization lateral projection confirming approximately 85% reduction in AVM nidus opacification with preservation of the major cerebral arteries. AVM, arteriovenous malformation

Pre-embolization DSA confirmed a 3-cm left parietal cerebral AVM (Spetzler-Martin Grade II) supplied predominantly by the superior division of the left middle cerebral artery, with an additional feeder arising from a middle cerebral artery perforator. Superficial venous drainage occurred through the vein of Trolard into the superior sagittal sinus (Figures 3A-3B). Single-session endovascular embolization under general anesthesia using the Lava 18 embolic agent achieved an estimated 85% angiographic reduction in nidus opacification on post-embolization angiography without procedural complications. Post-embolization DSA demonstrated marked reduction in the size and opacification of the AVM nidus with substantially reduced early arteriovenous shunting (Figures 3C-3D).

Given the favorable post-procedural recovery and complexity of the residual lesion, definitive microsurgical excision of the remaining nidus was planned as a staged procedure following neurological rehabilitation and outpatient reassessment.

No further seizure activity occurred following intervention. Neurological recovery gradually improved during the subsequent hospital stay. At discharge, seven days after endovascular embolization, the patient was hemodynamically stable, alert, and communicative with a Glasgow Coma Scale score of 15. Motor strength on the unaffected side remained preserved (5/5), while power on the affected side improved from 1/5 to 2/5. Expressive aphasia persisted at discharge; however, the patient was able to communicate using short phrases, gestures, and simple verbal responses and could participate in basic daily activities. She was discharged with advice for regular physiotherapy and follow-up after one month.

At one-month follow-up, the patient remained seizure-free and demonstrated continued neurological improvement. She was alert, communicative, and independent in basic daily activities. Speech had improved further, although mild expressive aphasia persisted. Lower limb power on the affected side improved from 1/5 during the acute phase to 5/5, allowing independent ambulation. Upper limb power improved from 1/5 to 3/5. No further neurological complications were reported.

## Discussion

Eclampsia is one of the most common causes of seizures during late pregnancy and the postpartum period. Because current diagnostic criteria do not require proteinuria, the initial diagnosis of presumed eclampsia was clinically appropriate in our patient, who presented with generalized seizures, impaired consciousness, and hypertension at 36 weeks of gestation [1]. However, when neurological recovery does not occur as expected after delivery, clinicians should broaden the differential diagnosis and investigate for other intracranial disorders.

Several neurological conditions can mimic eclampsia, including posterior reversible encephalopathy syndrome, cerebral venous sinus thrombosis, ischemic stroke, intracranial hemorrhage, and structural cerebrovascular lesions [2]. In our patient, persistent seizures and continued depressed consciousness despite emergency cesarean delivery were the first clues that the clinical course was atypical and warranted further evaluation.

Brain CT subsequently demonstrated a large left lobar intracerebral hemorrhage with marked mass effect and midline shift, fundamentally changing the diagnostic approach. In younger patients, lobar hemorrhage should prompt consideration of structural vascular lesions such as cerebral AVMs, aneurysms, or cavernous malformations rather than being attributed solely to hypertension [3]. Although emergency decompressive craniectomy and hematoma evacuation were lifesaving, the limited neurological recovery following surgery suggested that an underlying vascular lesion had not yet been identified.

Cerebral AVMs are uncommon vascular abnormalities and an important cause of hemorrhagic stroke in young adults [3,4]. Their estimated prevalence is approximately 10-18 per 100,000 individuals, corresponding to 0.01-0.02% of the general population [3]. Presentation during pregnancy is much less common, and many AVMs remain clinically silent until they present with hemorrhage or seizures [4].

Pregnancy-related intracranial hemorrhage is uncommon but carries substantial maternal morbidity and mortality [5]. Among women with known cerebral AVMs, the reported risk of hemorrhage during pregnancy is generally estimated at 2%-4% per pregnancy, although published data remain heterogeneous and the influence of pregnancy on AVM rupture continues to be debated [6]. Once rupture occurs, the risk of death and long-term neurological disability is considerable, emphasizing the need for early diagnosis and appropriate treatment [3,4].

An additional noteworthy aspect of this case is that the patient had completed two previous pregnancies without neurological complications, suggesting that the AVM had remained clinically silent for many years. Although a direct causal relationship cannot be established, pregnancy-related physiological changes, including increased blood volume and cardiac output, together with episodes of hypertension, may have contributed to rupture of the previously unrecognized lesion [4,6]. This illustrates the unpredictable natural history of cerebral AVMs and the diagnostic challenges they may pose during pregnancy.

Sequential use of non-contrast CT, CTA, and DSA was essential in identifying the underlying vascular lesion and planning treatment. DSA remains the reference standard for the diagnosis and characterization of cerebral AVMs and played a pivotal role in guiding endovascular management in our patient [7].

Previous reports have described cerebral AVMs presenting during pregnancy with headache, seizures, or intracranial hemorrhage and occasionally being mistaken for eclampsia [8-10]. In many of those cases, vascular imaging was obtained soon after neurological abnormalities became evident. In contrast, the diagnosis in our patient was established only after persistent neurological deterioration despite emergency cesarean delivery and subsequent decompressive craniotomy. This sequence highlights the importance of reconsidering the initial diagnosis when recovery after presumed eclampsia does not follow the expected course. Nevertheless, vascular imaging should be considered whenever feasible in young patients with spontaneous lobar intracerebral hemorrhage, regardless of subsequent neurological recovery.

The principal challenge in this case was not recognizing the AVM itself but distinguishing it from a far more common obstetric emergency. In many resource-limited healthcare settings, pregnant women presenting with seizures and hypertension are appropriately managed as presumed eclampsia because it is far more prevalent than intracranial vascular malformations. Under these circumstances, diagnostic anchoring is understandable. Nevertheless, persistent seizures, delayed recovery of consciousness, or unexplained focal neurological deficits after delivery should prompt urgent neuroimaging whenever feasible. In our patient, this change in diagnostic thinking ultimately led to identification of the ruptured AVM and definitive management.

Current treatment of cerebral AVMs often involves a multimodal approach incorporating microsurgical resection, endovascular embolization, and, in selected cases, stereotactic radiosurgery [7]. Our patient underwent staged treatment with endovascular embolization, achieving substantial reduction of the AVM nidus. Partial embolization was performed as part of a planned staged treatment strategy before definitive microsurgical excision. Because of the recent intracerebral hemorrhage and ongoing neurological recovery, definitive microsurgical excision of the residual nidus was planned after further rehabilitation and outpatient reassessment.

At one-month follow-up, the patient had regained independent ambulation, remained seizure-free, and demonstrated continued improvement in speech and upper-limb strength.

## Conclusions

This case highlights the importance of considering causes other than eclampsia in pregnant women who present with seizures and persistent neurological deficits. Non-contrast CT identified a life-threatening intracerebral hemorrhage and allowed emergency surgical evacuation. As the patient's neurological recovery did not follow the expected course, further evaluation with CTA and confirmatory DSA revealed a ruptured cerebral AVM, allowing endovascular embolization and planning for definitive treatment. Although vascular imaging in our patient was performed after persistent neurological deterioration, young patients with spontaneous lobar intracerebral hemorrhage should undergo vascular imaging whenever feasible to exclude an underlying vascular lesion. In settings where immediate access to advanced neurovascular imaging is limited, clinicians should maintain a high index of suspicion for underlying cerebrovascular disorders and arrange timely referral when the diagnosis remains uncertain or the clinical course is atypical.
